# Self-perception and clinical presentation of eating and swallowing difficulties within elderly care

**DOI:** 10.4102/sajcd.v72i1.1078

**Published:** 2025-03-04

**Authors:** Caitlin S. Bell, Esedra Krüger, Rouxjeanne Vermeulen, Andries Masenge, Bhavani S. Pillay

**Affiliations:** 1 Department of Speech-Language Pathology and Audiology, Faculty of Humanities, University of Pretoria, Tshwane, South Africa; 2 Department of Speech Language Pathology, School of Health Sciences, University of KwaZulu-Natal, Westville, South Africa; 3 Department of Statistics, Faculty of Natural and Agricultural Sciences, University of Pretoria, Tshwane, South Africa

**Keywords:** dysphagia, elderly, residential care facilities, self-perception, clinical assessment

## Abstract

**Background:**

The growing ageing population requires effective management of complex medical diagnoses and healthy ageing support within residential care facilities. However, limited access to guidelines on monitoring residents’ eating and swallowing abilities has been reported. Recent research is critical for future policy development.

**Objectives:**

This study aimed to compare self-perceived and clinical presentation of eating and swallowing abilities among a portion of elderly residents to enhance management of the residential care population within the South African context.

**Method:**

This comparative, within-subject research study assessed 44 participants using an oropharyngeal dysphagia protocol including a medical history review, the Eating Assessment Tool – 10 (EAT-10), the Mann Assessment of Swallowing Abilities (MASA), and the three-ounce water test of the Yale Swallow Protocol (YSP). A brief cognitive screener was used when cognitive impairment was unknown.

**Results:**

Of the participants, 21 out of 44 (48%) self-reported concerns for oropharyngeal dysphagia. Evidence of compensatory eating behaviours, without therapeutic intervention, was found. A negative, low correlation was present between the EAT-10 and the MASA (*r* = -0.306, *p* < 0.05) scores.

**Conclusion:**

Individuals who self-reported eating and swallowing difficulties demonstrated fewer clinical symptoms, potentially due to compensatory techniques. The disparity between patient-reported outcome measures and clinical assessment tools highlights the need for robust screening and assessment policies within this context.

**Contribution:**

This study highlights the importance of holistic assessment practices by integrating self-perception with clinical findings to address oropharyngeal dysphagia incidence within this complex population.

## Introduction

The United Nations (UN) estimates that by the year 2050, more than 2 billion people will be 60 years and older with much of this population residing in lower- and middle-income countries (United Nations, 2019). As the incidence of complex medical diagnoses increases with age, these evolving demographics pose a significant obstacle to delivering healthcare services capable of meeting the needs of the ageing population (The Organisation for Economic Co-operation and Development & World Health Organization, 2020). A mounting concern for the incidence of oropharyngeal dysphagia (OD) within this cohort is highlighted (Abu-Ghanem et al., 2020). With age-related physiological changes (such as loss of muscle mass and tissue elasticity, edentulism, xerostomia, and cervical spinal changes), combined with the burden of frailty and multi-comorbidity, the inherent risk of OD incidence increases (Ahn et al., 2020).

The World Health Organization (WHO) and European Union Geriatric Medicine Society (EuGMS) classify OD as a ‘geriatric syndrome’ thereby implying chronic, clinical impairment of the swallowing mechanism and associated decreased quality of life (Baijens et al., 2016; Geirsdóttir et al., 2021). The negative physiological sequelae of OD posing considerable danger to the elderly population include increased risk of malnutrition and dehydration, inability to swallow solid oral dosage forms of medication, frailty, asphyxiation, compromised respiratory status, lengthy hospitalisations, and ultimately fatality (Geirsdóttir et al., 2021; Logrippo et al., 2017). Clinicians are warned against overlooking the psychosocial impact of OD (Abu-Ghanem et al., 2020). Depression, anxiety, social isolation during mealtimes, feelings of loneliness, and periprandial stress are strongly associated with OD (Nishida et al., 2020).

Internationally, the provision of informal home-based care for ageing family members has declined because of caregivers’ insufficient clinical expertise and the rejection of traditional family structures that historically facilitated elderly care at home (Sun et al., 2021). The South African context has shown a mounting need for access to residential care facility (RCF) placement – trends have shown greater urbanisation from previously racially segregated areas and the rejection of historical cultural norms that is, caring for elderly members at home (Kalideen et al., 2022). This shift, combined with a longer life expectancy, underscores the demand for RCF placement within the South African context. The prevalence of OD in residents of RCFs is not fully known, yet previous studies indicate that OD is commonly overlooked as a typical part of the ageing process that results in this cohort being underdiagnosed and undertreated (Estupiñán Artiles et al., 2021; Logrippo et al., 2017). Inefficiencies within OD management are broadly observed within various healthcare settings. It is not uncommon for RCF staff to only refer to speech-language therapists (SLTs) for OD management when signs or symptoms of eating and swallowing difficulties become overt and are postulated to be inactive role-players in timeous identification of OD (Levenson & Walker, 2019).

A comparison of the self-perceptions and clinical presentation of eating and swallowing abilities of elderly residents within the RCF system is warranted. To encourage the provision of evidence-based healthcare, RCF staff require an informed understanding of the nature of OD presentation. This informed understanding of the presentation and incidence of OD within the local RCF system will not be possible without current research within the appropriate South African context. This study aimed to compare the self-reported perceptions and clinical presentation of eating and swallowing abilities within the elderly population residing in RCFs to potentially inform future policy that supports healthy ageing of this populous.

## Research methods and design

A total of 44 participants, with a mean age of 80 years (standard deviation = ± 6.54), were included in this sample. The data were collected from residents of pre-determined RCFs located in Johannesburg, South Africa. The RCFs that were selected were privately funded and were not associated with the Departments of Health or Social Development.

The ages of participants ranged between 65 years and 97 years. Potential participants had to be 65 years and older and permanently reside within the RCF to qualify for voluntary participation. Additionally, potential participants were excluded if they had received a formal diagnosis of moderate-to-severe cognitive impairment. Where the appropriateness of cognition was unknown to RCF staff, the Saint Louis University Mental Status (SLUMS) examination was administered (Morley & Tumosa, 2002). Only one prospective participant was excluded based on a previous diagnosis of moderate cognitive impairment.

A non-probability, purposive sampling strategy was used to recruit appropriate participants for this study (Brink & Van Rensburg, 2022). This sampling strategy was selected as it ensured participants included in the study were more likely to be predisposed to the phenomena being studied that is, OD is a geriatric syndrome with a high incidence rate among the RCF cohort (Baijens et al., 2016; Banda et al., 2022).

### Oropharyngeal dysphagia assessment protocol

**FIGURE 1:**
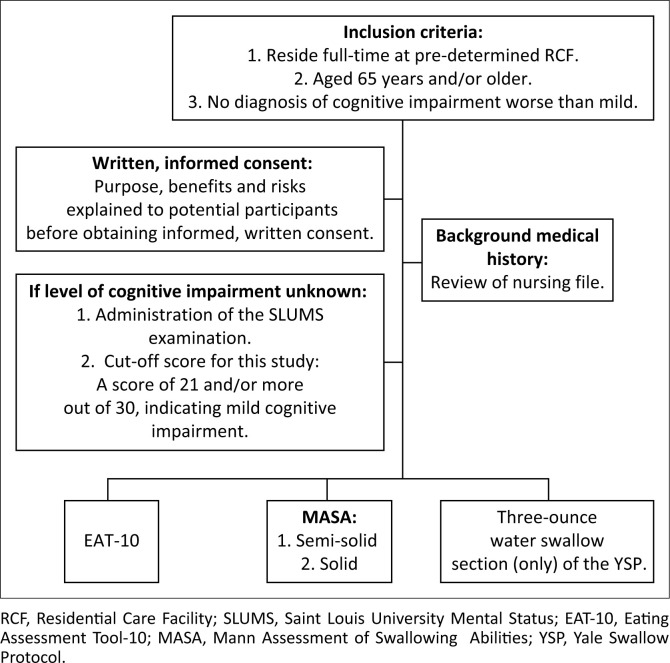
Oropharyngeal dysphagia protocol utilised during the research study.

A background case history form was utilised to identify demographic information, providing insight into the type of resident accommodation, level of autonomy during activities of daily living, medical history and current medical intervention received. Three standardised OD assessment tools were administered namely the Eating Assessment Tool – 10 (EAT-10), the Mann Assessment of Swallowing Ability (MASA), and the three-ounce water swallow challenge of the Yale Swallow Protocol (YSP) (Belafsky et al., 2008; Leder & Suiter, 2014; Mann, 2002). All evaluations were conducted during a single session. A second rater was used to ensure research rigour. Figure 1 outlines the OD assessment protocol guidelines utilised during this research study.

#### Eating assessment tool – 10

A patient-reported outcome measures (PROMs) tool was administered to establish participants’ self-perceptions regarding symptom-specific eating and swallowing concerns (Zhang et al., 2023). The EAT-10 demonstrates reliability and internal consistency when compared to patient outcomes of the Penetration Aspiration Scale, which is widely utilised in clinical practice for its ability to predict OD (Rosenbek et al., 1996). This tool is a self-administered, paper-based questionnaire including 10 statements rated from zero to four. For each statement, a score of zero indicates ‘no problem’ while a score of four would indicate ‘a severe problem’. The higher the final score out of 40, the higher the risk for aspiration because of impaired eating and swallowing. A total EAT-10 score between zero and two indicates no difficulty swallowing, while a score between 3 and 10 indicates a self-perceived swallowing difficulty.

#### Mann assessment of swallowing ability

This validated assessment tool was used to quantify the risk of aspiration and severity of possible dysphagia (Mann, 2002). The MASA tool utilises a 5-point to 10-point rating scale. The highest MASA score that can be achieved is 200 points, indicating normal swallowing physiology.

#### Three-ounce water swallow challenge of the Yale Swallow Protocol

The three-ounce water swallow challenge assessed participants’ ability to swallow three ounces of water (or approximately 90 mL) using sequential swallows and observing whether immediate or delayed signs of aspiration were present. This clinical screening tool was included in this study because of its strong negative predictive value (92.4%) when standardised in a prospective, double-blind and multi-rater study (Ward et al., 2020).

### Statistical analysis

The de-identified data were entered into Microsoft Office 2019. The statistical analysis system (SAS) version 9.4 was used to perform the analysis. On continuous variables, descriptive statistics such as means and standard deviations were applied, while frequency tables, that is, count and percentages were used on categorical variables. The Fisher’s exact test was used to determine the relationship between the outcome measures of the three-ounce water swallow challenge of the YSP and the EAT-10’s self-perceptions. The data were checked for normality using the Shapiro-Wilk test and found that the MASA and the EAT-10 scores were not normally distributed. The non-parametric test, Spearman’s rho correlation, was subsequently performed to determine whether the MASA and EAT-10 scores were correlated.

### Ethical considerations

Ethical clearance to conduct this study was obtained from the University of Pretoria Faculty of Humanities Research Ethics Committee (reference no. HUM039/0623). As encouraged by the Declaration of Helsinki, participation in this research study was voluntary and contingent upon receiving written informed consent, which was revokable (World Medical Association, 2013).

## Results

A total of 44 participants, with an average age of 80 years old (standard deviation ± 6.54), were included in this sample. Participants’ residential arrangements were predominantly reported as single-room configurations with individuals requiring support during eating and mealtimes. Only one participant was receiving speech-language therapy intervention for a diagnosis of OD because of ongoing oral cancer treatment at the time of the research study.

Only 1 out of 44 (2%) participants in the research study presented with no comorbidities and no prescribed chronic medication. Frequently identified comorbidities within the sample were high blood pressure (*n* = 28; 64%), depression and/or anxiety (*n* = 19; 43%), and thyroid disease (*n* = 12; 27%) (Table 1).

**TABLE 1:** A summary of participant reported comorbidities (*N* = 44).

Comorbidity	*n*	%
High blood pressure	28	64
Depression or anxiety	19	43
Thyroid disease	12	27
High cholesterol	11	25
Stroke	10	23
Diabetes	7	16
Osteoporosis	6	14
Heart disease	6	14
Heart failure	5	11
Arthritis	4	9
Parkinson’s disease	3	7
Cataracts	3	7
Emphysema	2	5
Asthma	2	5
Gout	2	5
None	1	2
Heart murmur	1	2
Coronary heart disease	1	2
Sciatica	1	2
Alcohol or substance abuse	1	2
Pneumonia	1	2
Hay fever and/or allergies	1	2
Frequent urinary tract infections	1	2
Kidney disease	1	2
Colitis	1	2

Among participants receiving chronic treatment for high blood pressure, the sensation of food sticking in the throat emerged as the predominant self-perceived eating and swallowing difficulty (*n* = 20; 45%). However, only three of these participants were clinically suspected of having OD when assessed using the MASA. A total of 22 out of 44 participants (50%) who were diagnosed with depression, anxiety, or thyroid diseases indicated additional effort taken to swallow pills on the self-perception scales of the EAT-10.

Numerous participants (*n* = 21; 48%) indicated an overall self-perceived concern for OD, indicating a mild-to-severe concern among the sample. The highest EAT-10 scores, presented in Table 2, were: (1) *swallowing pills takes extra effort* (*n* = 22; 50%) and (2) *when I swallow, food sticks in my throat* (*n* = 20; 45%). The lowest frequency of scores recorded, indicating no concern with OD, were: (1) *my swallowing problem has caused me to lose weight* (*n* = 12; 27%) and (2) *swallowing is stressful* (*n* = 8; 18%) (Table 2).

**TABLE 2:** A summary of participant rating on Eating Assessment Tool – 10 (EAT-10) (*N* = 44).

EAT-10 statement:	Participants with no concern		Participants with mild-to-severe concern	
	*n*	%	*n*	%
Swallowing is stressful	32	73	12	27
I cough when I eat	28	64	16	36
When I swallow, food sticks in my throat	24	55	20	45
Pleasure of eating is affected by my swallowing	31	70	13	30
Swallowing is painful	41	93	3	7
Swallowing pills takes extra effort	22	50	22	50
Swallowing solids takes extra effort	27	61	17	39
Swallowing liquids takes extra effort	31	70	13	30
My swallowing problem interferes with going out for meals	37	84	7	16
My swallowing problem has caused me to lose weight	32	72	12	27

EAT-10, Eating Assessment Tool – 10.

From the clinical swallow examination data, common clinical domains on the MASA that demonstrated an observable impairment were: (1) reduced tongue strength (*n* = 9; 20%); (2) diminished gag reflex (*n* = 14; 32%); (3) palatal reflex (*n* = 15; 4%); (4) poor bolus clearance (semi-solid *n* = 25; 57%) (solid *n* = 31; 70%); (5) lengthy oral transit time (semi-solid *n* = 21; 48%) (solid *n* = 20; 45%); (6) and the pharyngeal phase (semi-solid *n* = 10; 23%) (solid *n* = 11; 25%). For both a solid and semi-solid consistency, 41/44 participants (95%) scored above 177, thus indicating no established risk of OD and/or aspiration within this research study (Table 3).

**TABLE 3:** Frequency scores of the Mann Assessment of Swallowing Abilities (MASA) (*N* = 44).

Consistency assessed	Frequency score (%)					
	No abnormality		Mild risk		Moderate risk	
	*n*	%	*n*	%	*n*	%
**Dysphagia risk**						
Solids	41	93	2	5	1	2
Semi-solids	41	93	2	5	1	2
**Aspiration risk**						
Solids	42	95	1	2	1	2
Semi-solids	42	95	2	5	0	0

The administration of the three-ounce water swallow challenge demonstrated that 33 out of the 44 participants (75%) did not present with overt signs and/or symptoms of aspiration on a thin liquid. However, it should be noted that 20 of the 44 participants (45%) utilised a self-mediated compensatory swallow behaviour, that is, multiple swallows (instead of sequential swallows). Of the 11 of the total sample (25%) who failed the three-ounce water swallow challenge, less than half of these participants had previously indicated moderate-to-severe difficulty drinking thin liquids on the EAT-10 assessment tool (*n* = 5; 45%).

The Fisher’s exact test (*p* < 0.05) was applied to the outcome measures of the three-ounce water swallow challenge of the YSP and the self-perceptions of the EAT-10. An association between overt signs or symptoms of aspiration on thin liquids and the self-perceived additional effort taken to swallow a thin liquid was established. A statistically significant correlation between the absence of reported self-perceived difficulties in swallowing solid consistencies as assessed by the EAT-10 tool and the absence of clinical impairment in the pharyngeal response domain within the MASA (*p* = 0.02) was evident. The Spearman’s correlation coefficients were applied to the data evidencing a significant, negative low correlation between the MASA semi-solid score (*r* = 0.04, *p* < 0.05) and the EAT-10.

## Discussion

This research study revealed a varied disparity between the clinical presentation of OD and the reporting of self-perceptions regarding eating and swallowing abilities within the elderly population residing in an RCF. Despite a substantial portion of the sample indicating self-perceived concern for OD, the most frequent MASA score that was recorded highlighted no established risk of dysphagia and/or aspiration. Similar findings are described by an international study that analysed psychosocial wellness in the presence of OD (Leiman et al., 2023). The findings found that participants who did not demonstrate overt signs and/or symptoms of OD during clinical testing continued to explicitly report symptoms of eating and swallowing difficulties (Leiman et al., 2023).

A higher prevalence and/or perception of OD within the elderly could be associated with multimorbidity and polypharmacy, rather than ageing alone, with greater attention to swallowing function warranted in those with higher comorbidity indices (Da Silva et al., 2020; Jardine et al., 2021). Most of the research sample was treated for chronic multimorbidity, with a considerable portion of participants reporting a self-perceived eating and swallowing difficulty. Pre-existing chronic conditions such as hypertension, depression, anxiety, diabetes mellitus, previous stroke, cancer, and heart failure have been established as contributing factors to the manifestation of OD and were all present within this research sample (Abu-Ghanem et al., 2020; Banda et al., 2022).

Physiological changes clinically observed in this research study that correlated with previous studies include reduced lingual strength, diminished gag reflex, decreased palatal movement, incomplete bolus clearance, and delayed oral transit time (Mancopes et al., 2021). While the correspondence between self-reported OD concerns and the clinical presentation thereof varied, domains of clinically impaired physiology were commonly reported as self-perceptions of (1) *swallowing pills takes extra effort* and (2) *when I swallow, food sticks in my throat*. The most common comorbidities were hypertension, anxiety and/or depression, and thyroid diseases, all of which have a previously determined association with the manifestation of OD (Da Silva et al., 2020). The challenge posed to RCF staff lies in monitoring these subtle physiological changes because of chronic multimorbidity, widespread prescription of polypharmacy and unique variability in the incidence of OD within this cohort (Zanetti et al., 2023).

Consistent with other studies, this research study found that patients’ subjective experiences of eating and swallowing abilities can differ from the clinical findings of healthcare providers (Cohen & Hula, 2020; Zanetti et al., 2023). It was found that individuals who perceive difficulties with eating and swallowing potentially exhibit fewer observable clinical symptoms because of self-mediated compensations, that is, double-effortful swallows, paced and controlled boluses.

Conflicting findings were evidenced when comparing the results of a standardised tool, that is, three-ounce water swallow challenge of the YSP and the results of the PROM tool, that is, EAT-10. While an association between overt aspiration on thin liquids and the self-perceived additional effort in swallowing was established, the incidence of an aspiration event was reduced. The incidence in aspiration events could be influenced by the self-mediated compensatory swallow manoeuvres, such as paced, volume-controlled multiple swallows, instead of the prescribed sequential swallows used by participants. These compensations were not prescribed by a treating SLT and potentially indicate the participants’ insight into the adjustments that are necessary to maintain the safety and efficacy of eating and swallowing (Ahn et al., 2020; Mancopes et al., 2021; Wolf et al., 2021). Comparative literature that explores this relationship between self-mediated swallowing strategies and the reporting of self-perceived OD appears to be limited.

Swallowing difficulties should not be overlooked and attributed to age-related changes as this could undermine the timeous provision of healthcare referrals to specialised services for the RCF resident (Ahn et al., 2020; Estupiñán Artiles et al., 2021). A shift from the traditional biomedical model and alignment towards the consideration of patient self-perceptions could prevent the under-identification and under-treatment of eating and swallowing difficulties within the elderly population of the RCF system (Cohen & Hula, 2020).

### Limitations and future research

The sample size was relatively small and drawn exclusively from pre-determined, private RCFs impacting the extrapolation of the results to the broader population within different socioeconomic contexts of South Africa. Longitudinal observation and assessment during mealtimes could provide deeper insight into the frequency, type and indication for using self-mediated swallow manoeuvres within the RCF cohort. Analysis of these mealtime behaviours (or lack thereof) could further inform educational efforts with nursing staff to ensure that OD is not under-identified or misdiagnosed as a typical part of ageing.

## Conclusion

Reporting patterns for OD and the clinical presentation of symptoms within these local RCF systems were nuanced and varied. It is unlikely that the utilisation of one clinical tool supports the establishment of a holistic profile of eating and swallowing abilities but rather, consideration of stakeholder self-perceptions of OD could enhance assessment findings and inform treatment planning. Through the multidisciplinary team lens, including a SLT’s consultation during RCF policy planning is the foundational step in alerting RCF systems to the risks of inadequate OD management. To ensure improved OD monitoring and treatment practices within the South African RCF setting, the SLT is encouraged to engage in colleague education and role clarification with RCF nursing staff to improve the understanding and heighten awareness of OD. This improved monitoring and potential identification of OD by nursing staff could support timeous referrals and avoid overloading speech-language therapy caseloads in countries where the patient-to-therapist ratio is high.
